# The analysis of genome composition and codon bias reveals distinctive patterns between avian and mammalian circoviruses which suggest a potential recombinant origin for *Porcine circovirus 3*

**DOI:** 10.1371/journal.pone.0199950

**Published:** 2018-06-29

**Authors:** Giovanni Franzo, Joaquim Segales, Claudia Maria Tucciarone, Mattia Cecchinato, Michele Drigo

**Affiliations:** 1 Department of Animal Medicine, Production and Health (MAPS), University of Padua, Legnaro, Padua, Italy; 2 Departament de Sanitat i Anatomia Animals, Universitat Autònoma de Barcelona, Bellaterra, Barcelona, Spain; 3 UAB, Centre de Recerca en Sanitat Animal (CReSA, IRTA- UAB), Campus de la Universitat Autònoma de Barcelona, Bellaterra, Barcelona, Spain; Oklahoma State University, UNITED STATES

## Abstract

Members of the genus *Circovirus* are host-specific viruses, which are totally dependent on cell machinery for their replication. Consequently, certain mimicry of the host genome features is expected to maximize cellular replicative system exploitation and minimize the recognition by the innate immune system. In the present study, the analysis of several genome composition and codon bias parameters of circoviruses infecting avian and mammalian species demonstrated the presence of quite distinctive patterns between the two groups. Remarkably, a higher deviation from the expected values based only on mutational patterns was observed for mammalian circoviruses both at dinucleotide and codon levels. Accordingly, a stronger selective pressure was estimated to shape the genome of mammalian circoviruses, particularly in the *Cap* encoding gene, compared to avian circoviruses. These differences could be attributed to different physiological and immunological features of the two host classes and suggest a trade-off between a tendency to optimize the capsid protein translation while minimizing the recognition of the genome and the transcript molecules. Interestingly, the recently identified *Porcine circovirus 3* (PCV-3) had an intermediate pattern in terms of genome composition and codon bias. Particularly, its *Rep* gene appeared closely related to other mammalian circoviruses (especially bat circoviruses) while the *Cap* gene more closely resembled avian circoviruses. These evidences, coupled with the high selective forces apparently modelling the PCV-3 *Cap* gene composition, suggest the potential recombinant origin, followed or preceded by a host jump, of this virus.

## 1. Introduction

Several studies have demonstrated the presence of a relevant genomic signature in dinucleotide frequencies in different organisms [1–3]. For example, TpA is broadly under-represented in eukaryotic chromosomes, potentially because of its low thermodynamic energy, the high degree of degradation of UpA dinucleotides by ribonucleases in mRNA [4], or the presence of TA as part of many regulatory signals and stop codons [4]. Similarly, CpG dinucleotide scarcity in vertebrates is thought to be partially due to cytosine methylation. In fact, methylated cytosines are prone to spontaneous deamination to thymines, leading to the dinucleotide TpG [5] However, DNA conformation, such as secondary structure, and dinucleotide stacking energies can be involved in this bias [6].

Consequently, besides genomic structural constraints and chemical features, other factors affecting the genome stability, like environmental conditions (e.g. pH, temperature, metal concentration, etc.), can be involved in shaping the overall dinucleotide composition [1].

A similar signature pattern has been observed in term of codon usage. Due to the degeneracy of the genetic code, the 20 amino-acids are actually coded by 61 codons. However, the frequencies of synonymous codon usage appear non-random and different species exhibit more or less marked preferences [7]. Two non-conflicting hypotheses have been advocated to explain this scenario: the mutational (or neutral) and the selectionist hypotheses. The first one poses that the codon usage bias is ascribable to the genome composition and mutational patterns non-randomness. While some studies have actually demonstrated that the level of GC content (and more generally the genome composition) can explain part of the codon bias differences between different organisms [8,9], there are some clear evidences that natural selection must also be involved [7]. Supporting the selectionist hypothesis, the codon choice has been linked to translational levels, efficiency and accuracy. A role as an additional level of regulation, tuning the levels of protein abundance and their appropriate folding, has also been proposed [10–12]. Remarkably, a direct effect on organism fitness has been experimentally proven [13]. The concomitant action of these two forces, the so called ‘‘mutation-selection-drift balance model of codon bias” is currently the most commonly accepted theory explaining the codon bias, suggesting the selection favouring the most preferred codons and the mutational drift allowing the maintenance of the minor ones [7,14].

Based on these premises, a similar dinucleotide pattern between obliged intracellular parasites, like viruses, and their respective host can be expected. Selective forces should act favouring those individuals mimicking the host genomic composition to maximize the exploitation of the host cell machinery while minimizing at the same time the recognition by the defence system [15]. Moreover, parasites necessarily share the same environmental conditions of the host, thus it can be hypothesized that comparable forces act on both genomes. Surprisingly, the analysis of 86 viromes and microbiomes revealed that dinucleotide frequencies could allow an effective clustering of the biome based on its origin, suggesting that the environment is actually acting by selecting, directly or indirectly (i.e. favouring a limited number of dominant microorganisms), specific genomic pattern [16]. Similarly, a relation between virus and host genome has been demonstrated by several authors [17,18] and evidences of viral codon bias adaptation to the host one after host jump have been reported [19,20].

*Circovirus* genus includes species characterized by a monopartite, circular, ssDNA genome of about 1800 to 2000bp. Despite certain among-species variability, two main proteins are encoded in the viral genome: the *Rep* protein, involved in the host DNA polymerase mediated rolling circle replication, and the *Cap* one, constituting the viral capsid [21].

Despite the fact that this genus was recognized during the 70s [22,23], its clinical relevance was limited to avian species until the beginning of the nineties; *Beak and feather disease virus* (BFDV), *Pigeon circovirus* (PiCV) and *Goose circovirus* (GoCV) were already known as responsible for relevant diseases, but of marginal economic relevance [24]. It was with the emergence of the *Porcine circovirus 2* (PCV-2) that this genus rose as one of the major concerns for veterinary medicine. Due to the advances in diagnostic and sequencing techniques, members of *Circovirus* genus have been described in several animal species and the number of recognized species has currently increased to 29 species [25]; However,their clinical relevance is often unknown or negligible [26]. The aim of the present study was to investigate the features of dinucleotide and codon bias patterns in mammalian and avian viruses of the genus *Circovirus* to assess a potential association with the host tropism. Even if dedicated studies on codon usage have been published on some *Circovirus* species [27–29], no comprehensive comparative analysis relating these viruses with the respective host has currently been performed.

In 2016, a new porcine circovirus, tentatively named *Porcine circovirus 3* (PCV-3), was discovered [30] and found in tissues or serum of pigs suffering from different clinical conditions [30–33]; moreover, PCV-3 has also been detected in healthy animals [34]. Therefore, it is still too early to assess if PCV-3 is able to cause disease or not [35]. Interestingly, this new species appears distantly related to all known circoviruses and display, particularly in the capsid gene, a comparable amino-acid distance with mammalian (PCV-2) and avian (DuCV) infecting viruses [30]. Consequently, as a secondary study objective, specific analyses based on viral genomic feature evaluation were performed to provide further insights into the origin of PCV-3.

## 2. Materials and methods

### 2.1 Dataset

The whole collection of virus sequences classified into the genus *Circovirus* was downloaded from the NCBI Taxonomy browser (accessed 15/10/2017). In-house developed Python scripts were used for gene and features extraction, benefiting from the *Biopython* library functions [36].

*Rep* and *Cap* coding sequences were selected if their length was greater than 150 codons and non-terminal stop codon, undetermined nucleotides and out of frame mutations were absent. For each viral strain the sequence was extracted and annotated with the following metadata: accession number, viral and host species, country and date of collection. To homogenize the nomenclature, the reported host name was substituted by the scientific name. Additionally, the Class, Order, Family and Genus of the host were obtained through the NCBI Taxonomy and added to the previously described metadata.

### 2.2 Viral genome composition analysis

For each sequence the following statistics were obtained: content of each nucleotide (in percentage), total GC content (GC) and in codon positions 1 (GC1), 2 (GC2) and 3 (GC3).

The presence of a statistically significant difference among considered groups was evaluated using the Kruskal-Wallis test followed by Mann-Whitney Test with Bonferroni correction. The significance level was set to p<0.01.

The *rho* statistic was computed for each dinucleotide pair using the R library *seqinr* [37]. Briefly, the *rho* is the frequency of dinucleotide (*xy)* divided by the product of frequencies of nucleotide (*x)* and nucleotide (*y)* and it is expected to be equal to 1.00 when dinucleotide (*xy)* is formed by chance. To evaluate if some dinucleotide pairs were significantly over- or under-represented, a Z-score was calculated. The Z statistic is the normalization of the *rho* statistic by its expectation and variance according to a given random sequence generation model (i.e. nucleotide bases shuffling with replacement in their respective codon position).

### 2.3 Relative synonymous codon usage (RSCU) and effective number of codons (Nc)

The RSCU was calculated using the *seqinr* package in R. This statistic, indicative of codon bias, is calculated based on the number of times a particular codon is observed, relative to the number of times that the codon would be observed assuming a uniform synonymous codon usage. Consequently, the expected value is 1 in absence of any codon bias while synonymous codons with values lower than 0.6 or greater than 1.6 are regarded as under or over-represented, respectively [19,38].

The Nc values were calculated using the ENCPrime program [39,40]. This summary statistic represents the total number of different codons used in a sequence and can thus range between 21 (only one codon used for each amino-acid) and 60 (all synonymous codons are uniformly used) [12]. A second parameter, the Ncp statistic, also ranging between 21 and 60, was calculated to account for the effect of genome composition on codon bias [12,39]. Obtained Nc and Ncp values were plotted against their GC3 content and compared with the expected Nc distribution under the assumption that it is determined only by GC3 content.

### 2.4 Neutrality plot

The GC content in the first two codon positions (GC12) of each sequence was plotted against the respective GC3 content and the corresponding linear regression was estimated. This analysis aimed to evaluate the influence of mutational pressure and natural selection on codon usage patterns. If a statistical association would be demonstrated between GC12 and GC3, and the regression coefficient is close to 1, the mutational bias is assumed to be the predominant force driving the codon bias patterns. On the contrary, a regression slope close to 0 suggests the presence of selective pressure acting on and shaping the codon bias evolution. In this sense, the regression coefficient can be interpreted as a quantitative measure of the mutation-selection equilibrium [41–43].

### 2.5 Principal component analysis (PCA) and hierarchical clustering

The principal component analysis [44] was performed on the RSCU values, after centring and scaling, of the *Cap* and *Rep* gene datasets independently, using the *prcomp* function of the *stats* library in R [45]. The same approach was used selecting the dinucleotide *rho* statistics as variables.

Similarly, a hierarchical cluster analysis was performed on the same databases (i.e. RSCU and *rho* values) using a correlation-based dissimilarity distance. Briefly, the correlation among considered variable profiles was calculated for each sequence pair and converted in a dissimilarity measure (1-cor*(X)[j*,*k])*, where *j and k are the j*'th and *k*'th object (i.e. viral strain). The hierarchical clustering was calculated using as agglomerative method an *average* linkage using the *hclust* function of the *stats* library.

### 2.6 Host class prediction

Two different methods were developed to predict the taxonomic class of the infected host based on the viral genome composition (i.e. *rho* and RSCU). Particularly, a Linear Discriminant Analysis (LDA) and a Random Forest (RF) analysis were validated and their discriminatory capabilities were assessed calculating the Accuracy and the Cohen-K coefficient using a 10 fold cross-validation approach. Since the understanding of PCV-3 origin was one of the aims of the study, PCV-3 sequences were excluded from the training datasets and used only during the host class prediction step. All analyses were performed using the library *caret* and the relative dependencies [46].

## 3. Results

### 3.1Datasets

A total of 2555 *Rep* and 4424 *Cap* sequences were included in the final dataset. A complete list of the accession numbers and related information (e.g. virus species, host taxonomy, etc.) are provided as S1 Data.

Unfortunately, the limited number of strains collected from host classes different from *Aves* and *Mammalia* precluded the execution of meaningful comparisons. Consequently, unless otherwise stated, the analyses were focused on the circoviruses infecting mammals and birds.

### 3.2 Genome composition

**Fig 1:**
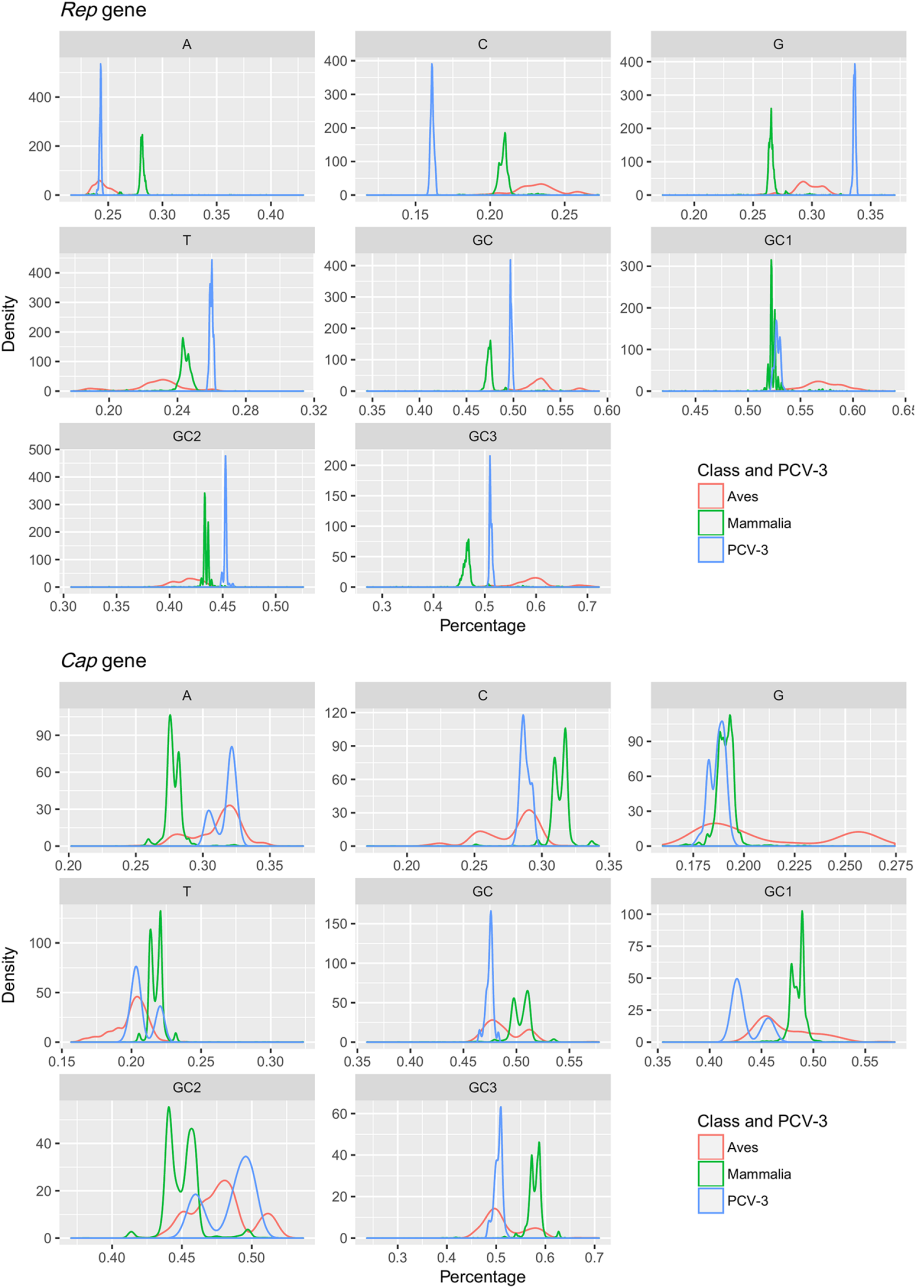
Circoviruses genome composition parameters. Density plot of the different genome composition parameters colour coded accordingly with the specific class category (i.e. Aves: 705 *Cap* and 933 *Rep*; Mammalia: 3705 *Cap* and 1601 *Rep*). PCV-3 (111 *Cap* and *40 Rep*) has been reported in blue. Both *Rep* (top) and *Cap* (bottom) genes have been analysed.

In the *Rep* gene, a statistically significant difference was demonstrated between circoviruses infecting different hosts in the mean value of all considered parameters (p-value<0.001). Globally, PCV-3 demonstrated a quite distinct pattern, being more closely related to avian or mammalian infecting viruses depending on the specific parameter studied (Fig 1). The only exception was represented by the A content (p-value = 0.82), where no significant differences were present with respect to circoviruses infecting the *Aves* class.

Although a significant diversity was demonstrated between avian and mammalian infecting viruses in the *Cap* gene (p-value<0.001), the PCV-3 genome composition appeared globally overlapping with the one of avian circoviruses (Fig 1). No statistically significant differences were found between these and PCV-3 in A (p-value = 0.019), C (p-value = 0.043) and GC3 content (p-value = 0.78).

Comparable results were obtained evaluating the dinucleotide composition. A less evident distinction was evident in the *Rep* gene a between circoviruses infecting *Aves* and *Mammalia* and among PCV-3 and the other two viral groups (S1 Fig). On the other hand, a clearer resemblance between PCV-3 and the avian circoviruses was evident in the capsid gene for practically all dinucleotide pairs (S1 Fig). The Z-score calculation in the *Rep* gene evidenced no under- or over-represented dinucleotide pair with the following exception: CpC, GpA and TpG were over-represented in mammalian infecting circoviruses while the CpG and TpC were under-represented in mammalian and avian circoviruses, respectively. CpG and GpG were slightly under- and over-represented in the PCV-3 *Rep* gene (S2 Fig). In the Cap gene, only mammalian circoviruses showed a significant deviation of dinucleotide frequency from what was expected; particularly, ApA, CpC, CpT and TpG were significantly over-represented while CpG and TpC were under-represented. Remarkably, a stronger bias was observed in the *Cap* gene compared to the *Rep* one. The TpT pair was the only dinucleotide over-represented in PCV-3 (S2 Fig).

### 3.3 Codon usage

Codon usage analysis showed a globally lower bias in the *Rep* gene compared with the *Cap* gene. The codons with a RSCU <0.6 or >1.6 are reported in Table 1 and S3 Fig. Briefly, 8, 9 and 14 codons were under-represented in the *Rep* of viruses infecting *Aves*, *Mammalia* and in PCV-3 while 4, 8 and 9 were over-represented, respectively. In the *Cap* gene, 9, 14 and 19 codons were under-represented in viruses infecting *Aves*, *Mammalia* and in PCV-3 while 7, 9 and 12 were over-represented, respectively. PCV-3 had a globally higher bias in several codons; however, the remarkably lower number of available sequences increased the likelihood of more extreme values.

**Table 1:** Over- and under-represented codons.

Group	Type	*Rep gene*	*Cap gene*
**Aves**	Under-represented	aca,agg,ata,cta,gca,gta,tca,tta	acg,cat,cgg,ctt,gag,gct,ggg,ggt,tgt,
	Over-represented	acc,agc,atc,ctg	aga,agc,cca,gaa,gca
**Mammals**	Under-represented	acc,ccg,cgt,cta,gca,gcc,gcg,tca,tta	acg,agc,ccg,cga,cgg,gag,gca,ggg, ggt,tca,tct,tgt,ttg
	Over-represented	aat,aga,agc,att,ctg,gct,tcc, ttg	acc,aga,ccc,cgc,ctc,ctt,gaa,ggc,tcc,tgc
**PCV-3**	Under-represented	aca,atc,cat,ccc,cga,cgc,cta,ctc,ctt,gac,ggc,gtc,tac,tca,tcc,ttc	aat,acg,agt,atc,cat,ccg,cga,cgg,gag, gat,ggg,ggt,gta,gtg,tca,tta,ttg
	Over-represented	agc,agg,att,cgg,ctg,gat,ggg,gtt,ttg	aac,aga,agc,att,cac,cgt,cta,ctc,gaa, gac,gga,gtt,tcc

Summary of over- and under-represented codons in the genes encoding the *Rep* and *Cap* proteins of avian and mammalian circoviruses and PCV-3.

### 3.4 Nc plot

**Fig 2:**
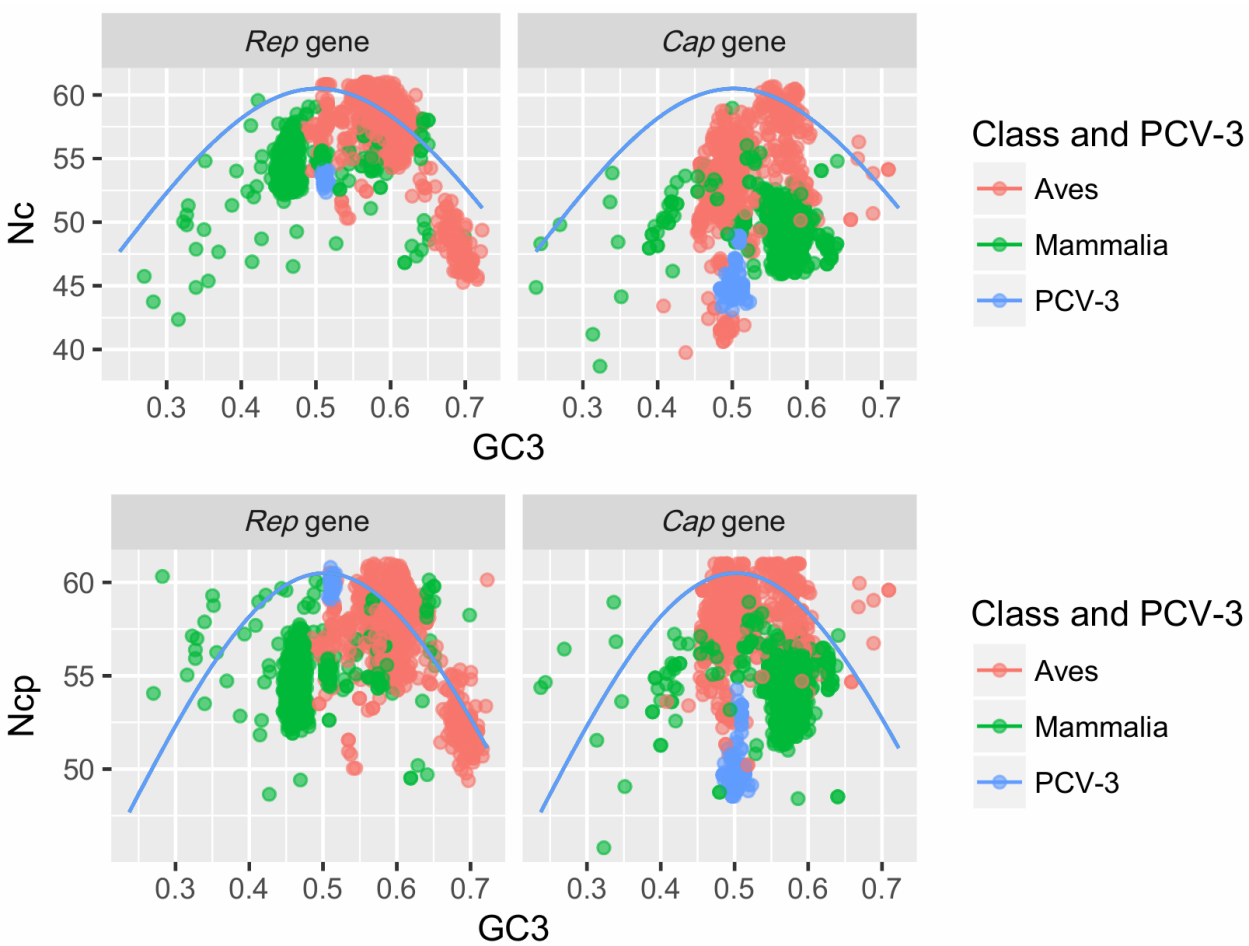
Nc and Ncp plot. Scatterplot reporting the relationship between Nc and Ncp and GC3 content for the *Rep* and *Cap* genes. Avian and Mammals circoviruses and PCV-3 have been color-coded. The line representing the expected Nc values, which would result from GC composition being the only factor influencing the codon usage bias, has been superimposed.

The RSCU results were substantially confirmed by the Nc analysis, being Nc values significantly (p-value<0.001) higher (i.e. lower codon bias) on average in the *Rep* than in *Cap* gene (p-value<0.001) and globally lower in mammalian infecting viruses compared to avian ones (p-value<0.001) (Fig 2). Circoviruses infecting mammals demonstrated a higher deviation of the Nc values from the expectation based on GC3 (S4 Fig). On the contrary, avian circoviruses Nc globally mimicked the expected pattern, at least for the *Rep* gene. Interestingly, when the background nucleotide composition was accounted, an overall reduction in the gap between expected and observed values was observed. This reduction was particularly evident for the Ncp value of PCV-3 in the *Rep* gene, which substantially moved to the expected range. Similarly, the Ncp values of the avian circoviruses overlapped the expected ones in the *Rep* gene, while a significant deviation was still present for some avian strains in the *Cap* gene and in both *Rep* and *Cap* genes of the *Mammalia* infecting circoviruses.

### 3.5 Neutrality plot

A statistically significant relationship between GC12 and GC3 was found in the *Rep* gene of avian circoviruses (b = 0.27; p-value<0.001), mammalian circoviruses (b = 0.47; p-value<0.001) but not in that of PCV-3 (p-value = 0.26), although a certain correlation seemed present (b = 0.21). Similar results were obtained for the *Cap* of avian circoviruses (b = 0.29; p-value <0.001) but not of mammalian circoviruses, which slope was remarkably lower (b = 0.13, p-value < 0.001), and of PCV-3, demonstrating no relationship between GC12 and GC3 (b = 0.02;p-value = 0.75). Consequently, the mutational drift accounted for about 25% of the codon bias in avian circoviruses, 40% and 15% in the *Rep* and *Cap* of mammalian circoviruses, and was negligible for PCV-3, although some evidences of its action were present in the *Rep* gene.

### 3.6 Principal component analysis and hierarchical clustering

**Fig 3:**
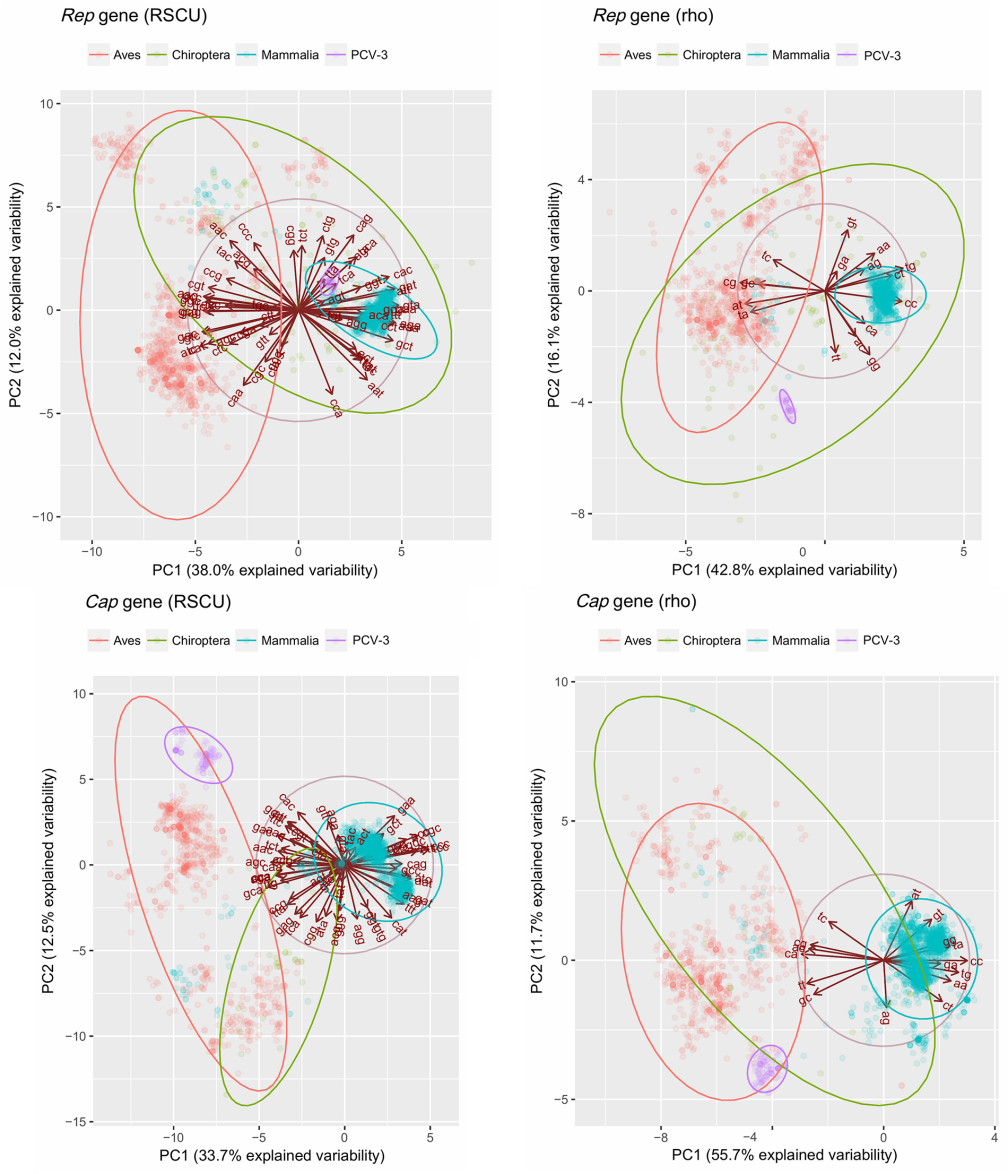
PCA based on RSCU and *rho* values. Scatter plot based on the first two components of the PCA performed on RSCU and *rho* values calculated on mammal and avian circoviruses. For interpretation easiness, PCV-3 and *Chiroptera* circoviruses have been highlighted with different colours. The PCA loading are represented as arrows. The 95% confidence ellipses around clusters are also reported. Both *Rep* (top) and *Cap* (bottom) genes have been analysed.

After PCA eigenvalues evaluation, the first two principal components (PC1 and PC2) were maintained for both *Rep* and *Cap* genes, since they explained a good percentage (i.e. always greater than 45%) of the observed variability. The avian and mammalian circoviruses formed, with few exceptions, easily separable groups when recoded using the PC1 and PC2 obtained from either the RSCU or the *rho* datasets. PCV-3 represented an interesting exception since it was related to mammalian viruses for the *Rep* gene, particularly in terms of codon bias, while it appeared more similar to avian infecting species for the *Cap* gene (Fig 3). Fully comparable results were obtained using the hierarchical clustering approach (S5 Fig). To investigate the possible relationship between PCV-3 and other circoviruses infecting hosts not belonging to the *Aves* and *Mammalia* classes, a hierarchical clustering was performed on the whole dataset, which included all the available host taxa. Nevertheless, PCV-3 always clustered with avian or mammalian circoviruses, in accordance with the patterns previously described (data not shown).

### 3.7 Predictive methods

**Fig 4:**
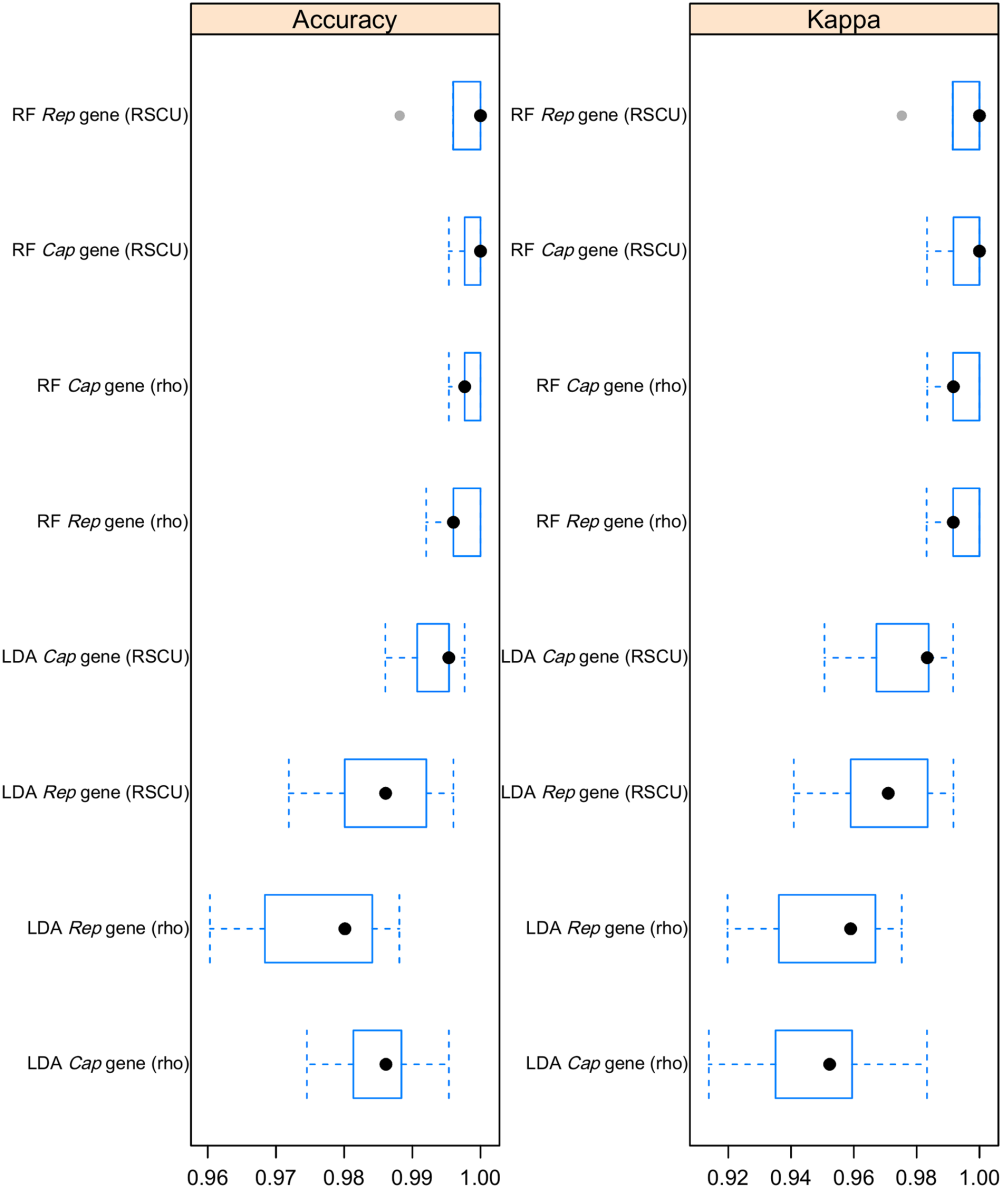
Diagnostic performances of predictive methods. Distribution of diagnostic performance metrics of RF and LDA evaluated by cross-validation on Cap and Rep datasets.

The two validated predictive models showed remarkable discriminative capabilities for both the *Rep* and *Cap* genes (Fig 4). However, when the host class was predicted for PCV-3 sequences, conflicting results were obtained between the two considered genes. In fact, the *Rep* gene was classified as “*Mammalia*” even with a relatively high degree of uncertainness, while the *Cap* gene was classified in the “*Aves*” infecting virus category (Table 2).

**Table 2:** Predictive method performances.

		*Rep gene*		*Cap gene*	
Dataset	Method	Class prediction	Probability (mean and range)	Class prediction	Probability (mean and range)
***rho***	LDA	Mammalia	0.99 (0.95–0.99)	Aves	1 (0.99–1)
	RF	Mammalia	0.62 (0.60–0.64)	Aves	1 (0.99–1)
**RSCU**	LDA	Mammalia	0.99 (0.99–1)	Aves	1 (0.99–1)
	RF	Mammalia	0.62 (0.59–0.65)	Aves	0.68 (0.61–0.73)

Results of PCV-3 host-class prediction performed using different datasets (i.e. *rho* statistic and RSCU) and predictive methods (i.e. LDA and RF). The most likely class and the estimated probability are reported for different method-dataset combinations. The probability range has been obtained by estimating the host-class using all the available PCV-3 genomes.

## 4. Discussion

Virus existence and maintenance lie on an intimate relationship with their host, since they depend on the same cell machinery, share the same physical and biochemical environment, and struggle with each other for survival. Consequently, viruses are expected to mirror (or at least be influenced by) the host genomic features, which have a huge impact on genome structure, RNA transcription and stability, protein translation and folding [11]. A marked adaptation to the “host environment” appears particularly realistic for ssDNA viruses since they totally depend on the host cell machinery for replication and are devoid of the panel of proteins used by other, more complex viruses, to interfere with the host immune response [47]. The results of the present study largely confirm this host-adaptation, since circoviruses infecting avian and mammalian species show a quite distinct pattern in terms of genome composition, dinucleotide frequency and codon bias.

Avian circoviruses show a globally higher C, G and particularly CpG content compared with mammalian ones, whose genome was proven to be deprived of these nucleotides. Remarkably, the avian genome does not differ significantly from the mammalian one with regard to CpG content and overall GC percentage [48]. Thus, other explanations to the different circovirus composition patterns must be claimed besides simple genomic mimicking. The fitness and spreading of ssDNA viruses depend on a rapid replication, anticipating the development of an effective host immune response [47]. Therefore, a high CpG dinucleotide content was proposed to be deleterious for these viruses since it can slow down the duplication and transcription processes because of the high stacking energy of CpG dinucleotide pair [47]. However, avian species typically exhibit a higher body temperature than mammals [49,50] and the different thermodynamic environment could provide enough free energy for an efficient replication, while the greater GC content would guarantee the stability of relevant secondary structure [51,52]. The deamination of cytosine to thymines has been proposed to explain the CpG under-representation in vertebrate genomes. However, the methylation of actively replicating virus genomes, although proven [53], is still a poorly documented phenomenon which frequency and relevance remain unknown [54].

Interestingly, a similar scenario was described for influenza A virus after its host jump from birds to human, leading to the 1918 pandemic. Since then, an overall decrease in CpG content of influenza viruses was observed, which has been attributed to an attempt to reduce the Toll-like receptor (TLR) (potentially TLR3, TLR7, TLR8 and RIG-I) viral recognition mediated by CpG motifs of the RNA molecules [15]. Remarkably, the ancestral 1918 influenza virus strain and modern avian derived strains appear to induce a more marked innate immune response [55]. A similar mechanism could be involved also in the different dinucleotide pattern observed in avian and mammalian circoviruses. In vertebrates, unmethylated CpG motifs are involved in the recognition of viral DNA genome mediated by the TLR-9. Interestingly, this TLR has been deleted by the avian genome [56] and no orthologue gene has been found [57]. In avian species, a comparable function is carried out by the TLR-21, which appears sensitive to the same motifs [57,58]. However, a differential activation has been reported between TLR-9 and TLR-21 when stimulated by pathogens [59,60] and, therefore, a different virus-host interaction may take place. If these physiological differences are actually responsible for differential evolutionary pressures acting on virus evolution, needs further investigation.

The *Cap* gene demonstrated a marked codon bias in avian and especially in mammalian circoviruses. In the latter, a relevant deviation from the expected Nc based on GC3 was observed. Accordingly, the neutrality plot analysis comes out on the side of a prominent action of natural selection on mammalian circovirus *Cap* codon bias, whereas mutational drift is more involved in the *Rep* gene evolution and, more generally, in the evolution of avian circoviruses as well. While an exhaustive explanation of the different patterns observed in the two animal classes is challenging, the evidence that evolution appears to be directed towards the selection of CpG depleted synonymous codons, particularly in highly expressed capsid protein, suggests a trade-off between a tendency to optimize the capsid protein translation while minimizing the recognition of the genome and the transcript molecules.

In 2016, a new porcine circovirus (PCV-3) was discovered in pigs. The recent identification and the low genetic diversity of the currently sequenced strains, which would suggest a recent PCV-3 origin, conflict with the absence of closely related circoviruses. Remarkably, the analysis of the genome composition, dinucleotide frequency and codon bias led to cluster the capsid gene of this virus together with avian circoviruses. On the contrary, a resemblance was observed between PCV-3 *Rep* gene and other mammalian circoviruses. These results were further confirmed by two independent classification methods that performed excellently on other known circoviruses. Although the development of host-prediction tools was beyond the scope of the present study, the accurate results provided by the two methods demonstrated that codon bias and genome composition were informative enough to predict the viral tropism and, indirectly, support the effect of host environment in shaping viral genome evolution. Surprisingly, the CpG content in the PCV-3 *Cap* gene substantially overlaps the one of avian circoviruses, which is in sharp contrast with the hypothesized role of CpG depletion in reducing mammal innate immunity activation. Moreover, while the PCV-3 *Rep* gene effective number of codons can be explained mainly by genome composition background (as shown in Fig 2), other forces appear to remarkably act on the *Cap* gene. Accordingly, the PCV-3 *Cap* gene was the only one where absolutely no correlation was demonstrated between GC12 and GC3 content. Therefore, the presence of a strong selective pressure shaping the PCV-3 *Cap* gene patterns can be confidently stated; this scenario is fully compatible with the recent introduction in a new environment (i.e. from avian to mammals species), as demonstrated for other viruses experiencing a recent host jump [15,20]. The role of recombination in the emergence of this new virus can therefore be suggested. In fact, although the clustering with mammalian circoviruses appeared globally weak, particularly at dinucleotide level, PCV-3 exhibited a rather surprising similarity with some bat circoviruses in the *Rep* region, either in codon usage and dinucleotide frequency. Members of the order *Chiroptera* are reservoirs of several viruses and are considered the source of many emerging diseases [61]. Many biological features enable them to carry a diversity of viruses. They represent about 20% of all mammalian species [62], hence providing a remarkable genetic heterogeneity. Since their ancient origin (about 52.5 million years ago), many viruses could have progressively co-evolved with bats [63]. Moreover, the absence of a bone marrow producing B cell as well as other peculiarities in the immune system (reviewed in Baker et al., 2013) [64] provide a favourable immune environment for viruses to survive and being maintained in these species [65]. Finally, their worldwide distribution, social behaviour and ability to fly guarantee advantageous conditions for the genesis of huge viral populations and their spreading [63]. Despite no clear evidences are available about the bat role as mixing vessels for avian and mammalian viruses, some data suggest their potential susceptibility to both types of viruses. Serological data have reported a seroprevalence of about 30% against avian influenza subtype H9 in Ghanaian bats [66] and little brown bats (*Myotis lucifugus*) were proven to co-express both avian and human type influenza receptors in their respiratory and gastrointestinal systems [67]. Remarkably, different species of bat circoviruses have different genome composition, ranging from mammalian- to avian-like (as shown in Fig 3). Therefore, the possibility to harbour genetically distant viruses could have favoured the emergence of recombination events. While these results can-not be automatically used to infer a bat role in PCV-3 emergence and the intrinsically poorly informative genetic data may hinder definitive conclusions, they at least support the plausibility of the offered hypotheses. Unfortunately, the knowledge of the virosphere is still at its infancy and the lack of information hampers both more precise identification of the bat role in avian-like virus evolution as well as the understanding of the PCV-3 origin.

In conclusion, the present study demonstrates the presence of quite distinctive patterns in genomic composition, dinucleotide frequency and codon bias between circoviruses infecting mammalian and avian species. Although several forces appears to be in place, including the mutational bias, a significant trade-off between the reduction of host innate immune response recognition and the maximization of translation efficacy, particularly of the capsid protein, seems to be the driving forces shaping circovirus genomic evolution. Moreover, the analysis of these parameters allowed to speculate a potential recombinant origin, followed (or preceded) by a host jump, of PCV-3. The genome of this virus appears to result from the combination of a mammalian-virus (likely a bat-circovirus) *Rep* gene with an avian circovirus-like *Cap* gene.

## Supporting information

S1 Data List of selected sequences and metadata. List of *Rep* and *Cap* sequences used in the study. Additional metadata including viral and host species taxonomy are reported.
(XLT)

S1 Fig Density plot of dinucleotide pairs. Density plot of the different dinucleotide pairs colour coded accordingly with the specific class category. PCV-3 has been highlighted in blue.
(PDF)

S2 Fig Z-score of dinucleotide pairs. The mean value (points) and 95CI (error-bars) of the Z-score for different dinucleotide pairs are reported and colour-coded according to the animal class. Both *Rep* (top) and *Cap* (bottom) genes have been analysed. Z-score higher and lower than 1.96 (i.e. statistically different from 0) have been highlighted by dotted lines.
(PDF)

S3 Fig Relative synonymous codon usage. The mean value (points) and 95CI (error-bars) of RSCU of the analyzed viral strains are reported and colour-coded according to the animal class. Both *Rep* (top) and *Cap* (bottom) genes have been analysed. The values corresponding to overrepresented and underrepresented codon thresholds have been reported as dotted lines.
(PDF)

S4 Fig Deviation of Nc values from expectation. Boxplot reporting the deviation of the *Rep* and *Cap* genes Nc values from the expectations based on GC3. Avian and Mammals circoviruses and PCV-3 have been colour-coded.
(PDF)

S5 Fig Hierarchical clustering. Hierarchical clustering obtained from *rho* and RSCU values of the *Rep* and *Cap* genes. The different animal groups have been colour coded. For graphical reasons, only a subset (i.e. a maximum of 5 randomly selected sequences for each viral species) is represented.
(PDF)
