# Supplementary figures and images for: The analysis of genome composition and codon bias reveals distinctive patterns between avian and mammalian circoviruses which suggest a potential recombinant origin for Porcine circovirus 3
Source: PLoS One. 2018 Jun 29;13(6):e0199950. doi: 10.1371/journal.pone.0199950 (PMC6025852; doi:10.1371/journal.pone.0199950)

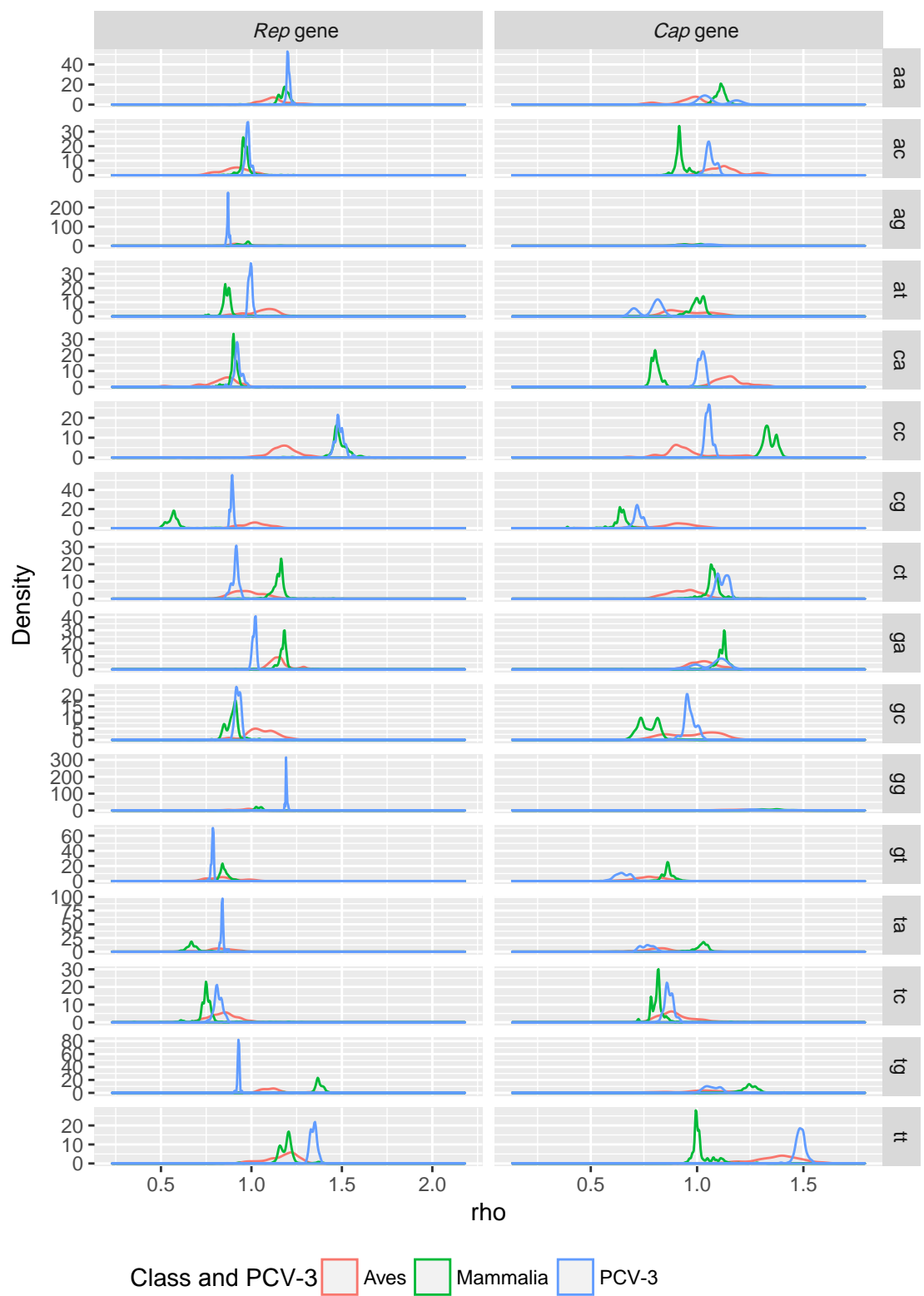

Supplement: S1 Fig — Density plot of the different dinucleotide pairs colour coded accordingly with the specific class category. PCV-3 has been highlighted in blue. (PDF) [file pone.0199950.s002.pdf]

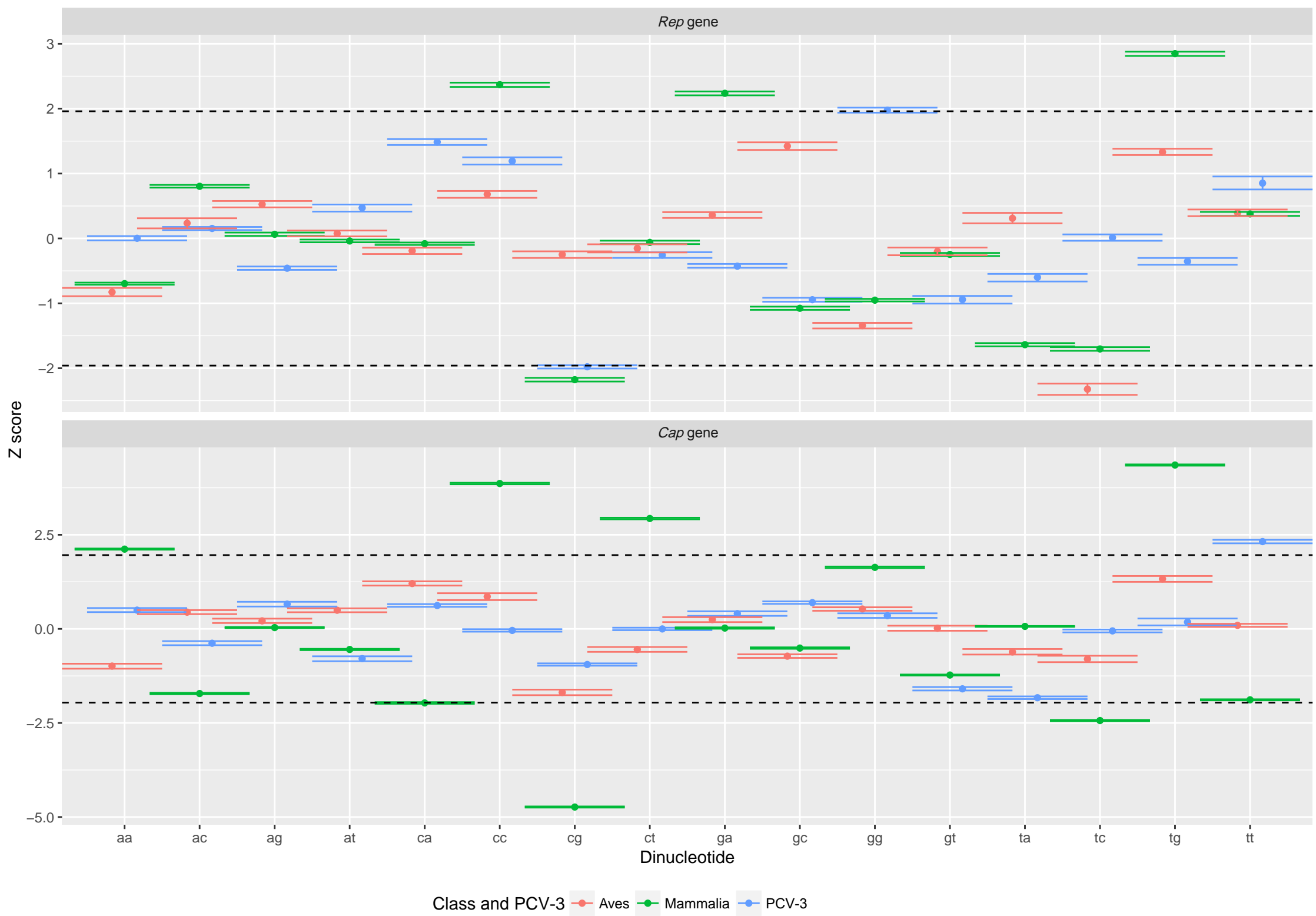

Supplement: S2 Fig — The mean value (points) and 95CI (error-bars) of the Z-score for different dinucleotide pairs are reported and colour-coded according to the animal class. Both Rep (top) and Cap (bottom) genes have been analysed. Z-score higher and lower than 1.96 (i.e. statistically different from 0) have been highlighted by dotted lines. (PDF) [file pone.0199950.s003.pdf]

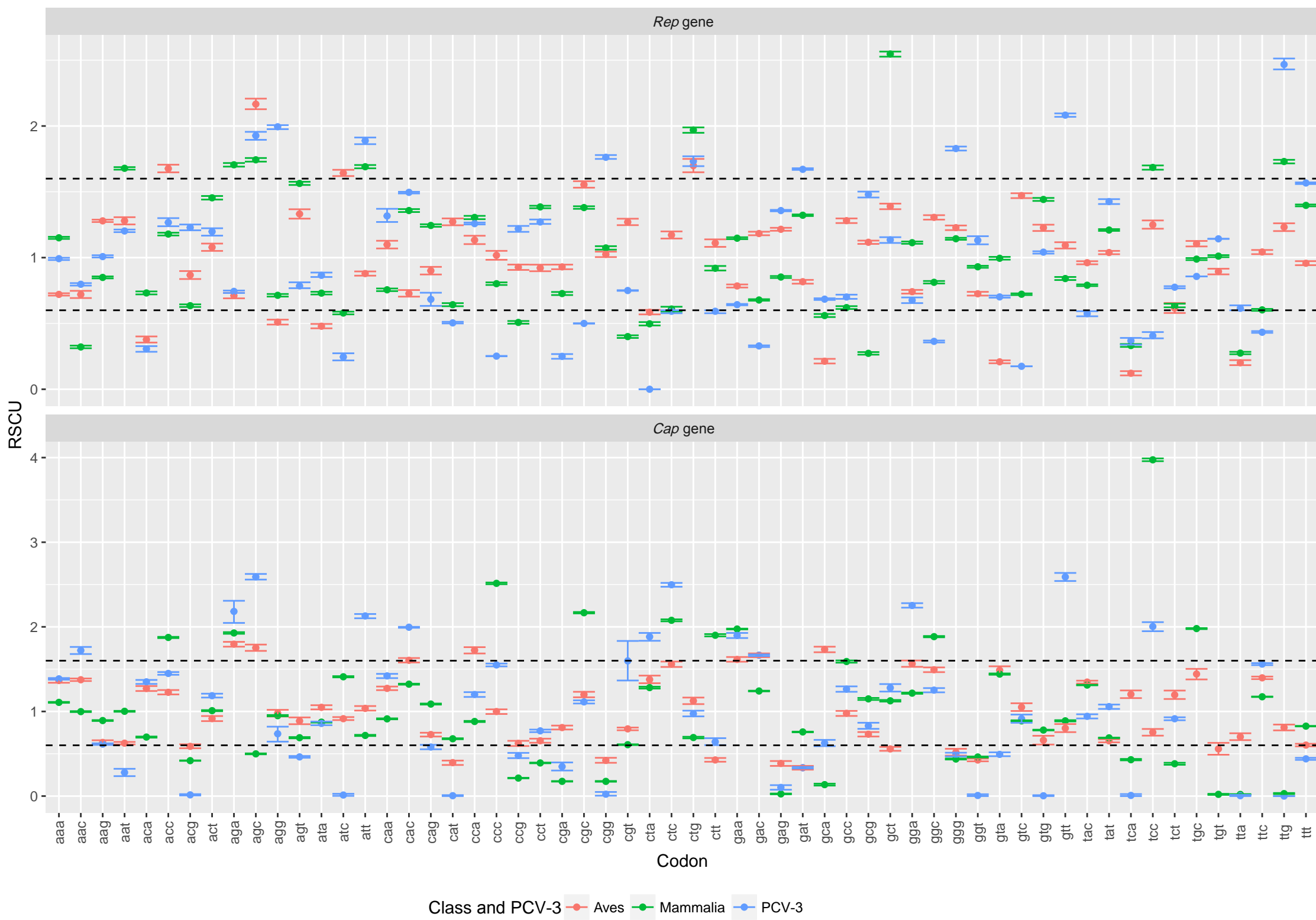

Supplement: S3 Fig — The mean value (points) and 95CI (error-bars) of RSCU of the analyzed viral strains are reported and colour-coded according to the animal class. Both Rep (top) and Cap (bottom) genes have been analysed. The values corresponding to overrepresented and underrepresented codon thresholds have been reported as dotted lines. (PDF) [file pone.0199950.s004.pdf]

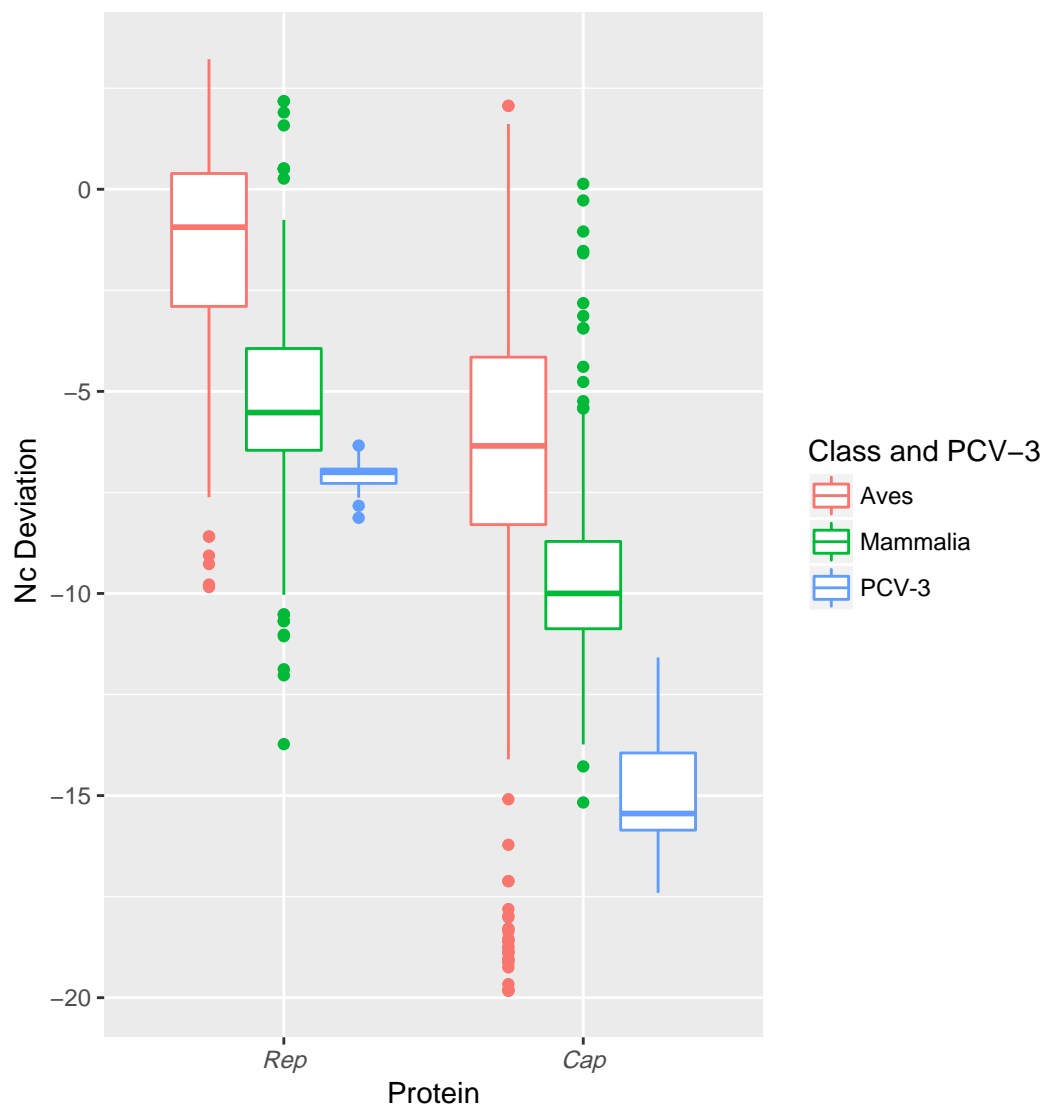

Supplement: S4 Fig — Boxplot reporting the deviation of the Rep and Cap genes Nc values from the expectations based on GC3. Avian and Mammals circoviruses and PCV-3 have been colour-coded. (PDF) [file pone.0199950.s005.pdf]

Rep gene (rho)

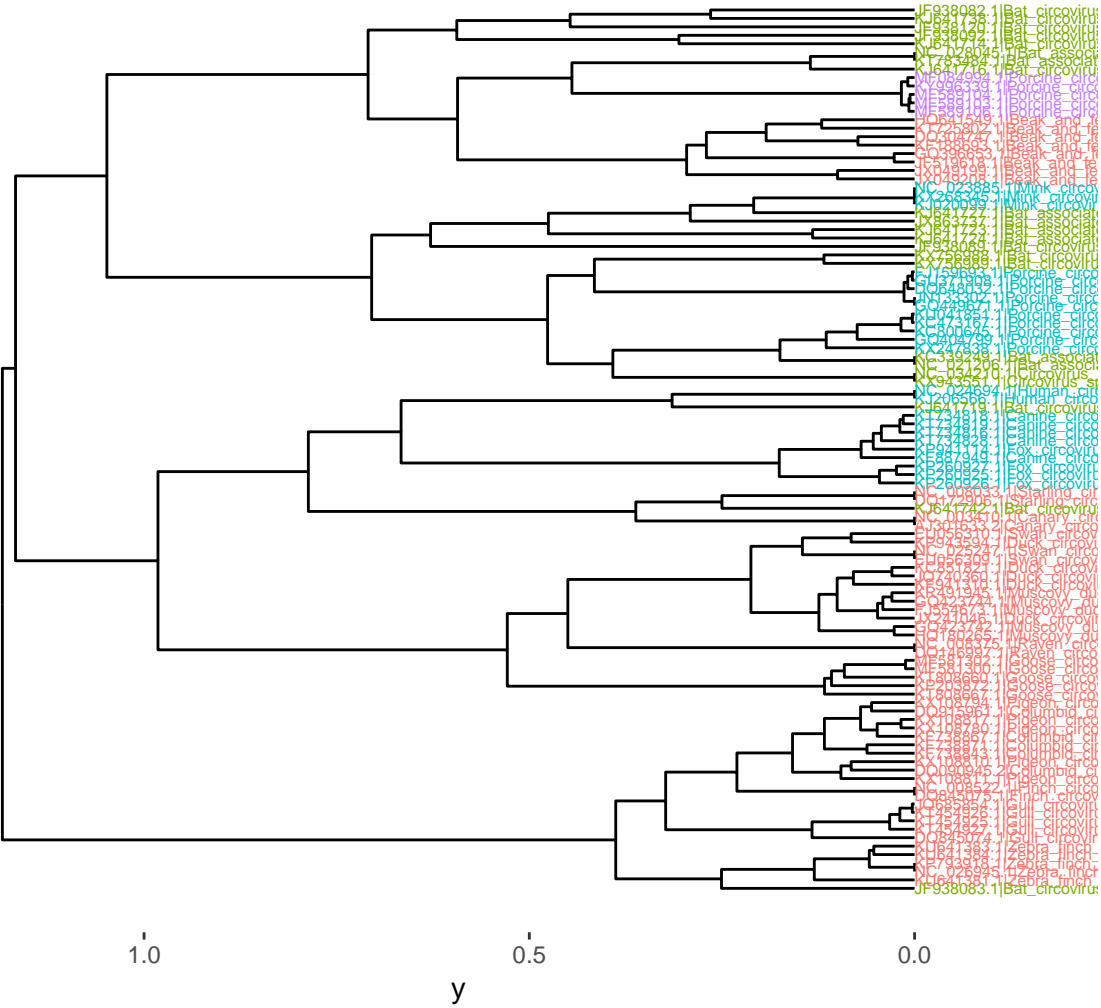

Class and PCV-3

- a Aves
- a Chiroptera
- a Mammalia
- a PCV-3



*Cap* gene (rho)

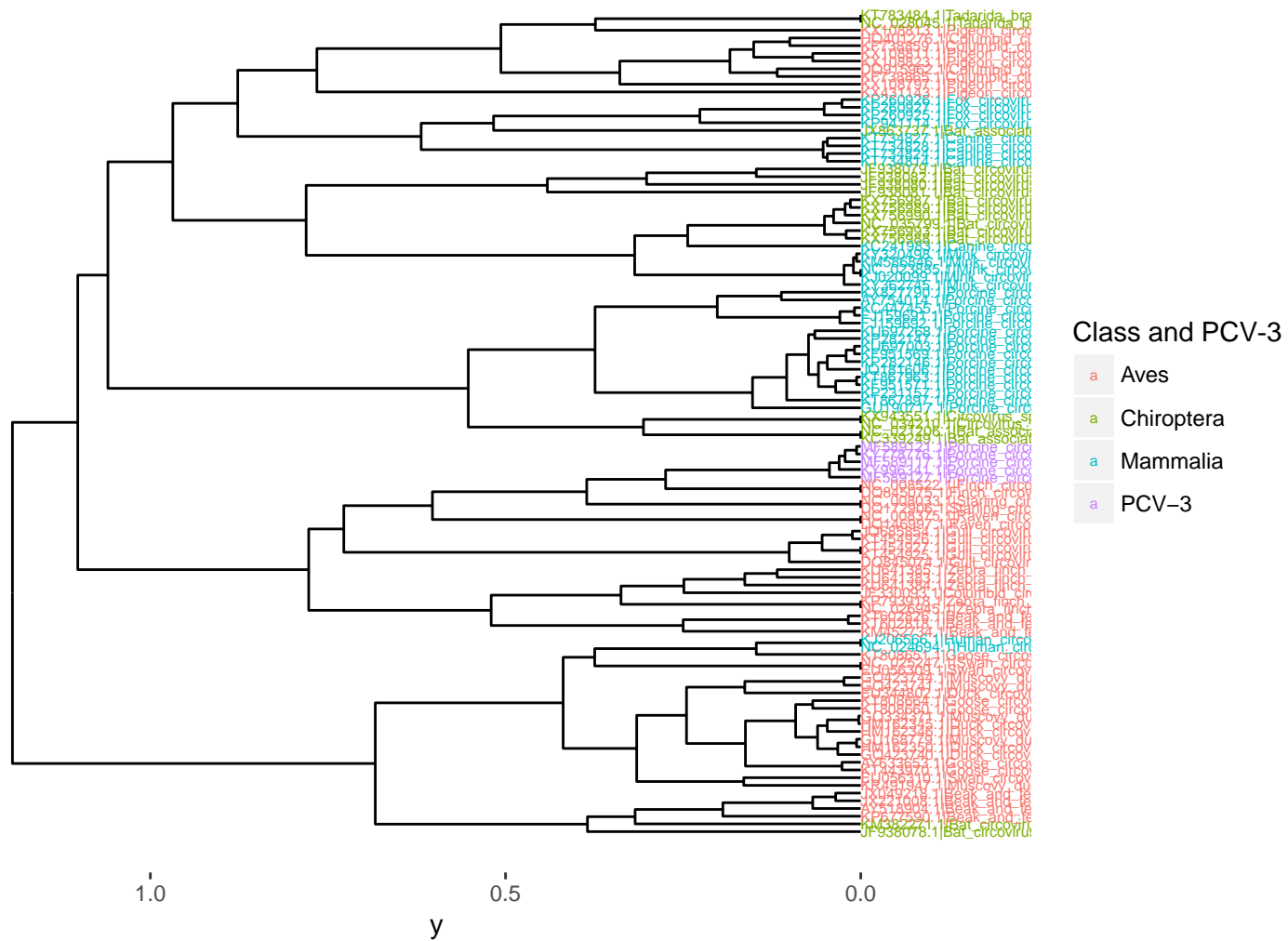

*Cap* gene (RSCU)

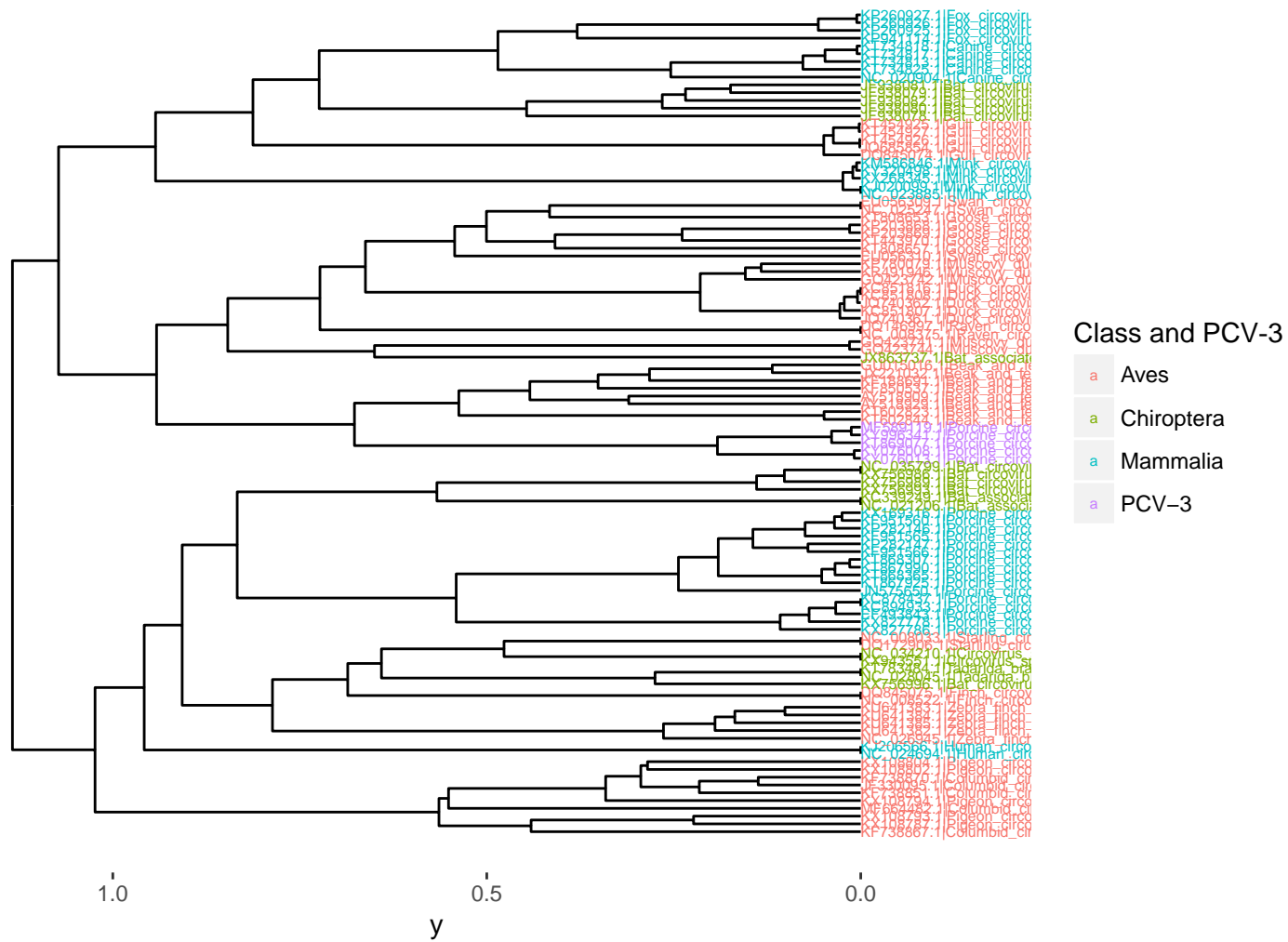

Supplement: S5 Fig — Hierarchical clustering obtained from rho and RSCU values of the Rep and Cap genes. The different animal groups have been colour coded. For graphical reasons, only a subset (i.e. a maximum of 5 randomly selected sequences for each viral species) is represented. (PDF) [file pone.0199950.s006.pdf]
